# The center of wheat domestication drives diversity of *Clavibacter* pathogens

**DOI:** 10.1128/aem.01245-25

**Published:** 2025-10-08

**Authors:** Asma Rahmanzadeh, S. Mohsen Taghavi, Sadegh Zarei, Hamid Abachi, Nastaran Zamani, Mozhde Hamidizade, Ardavan Soleimani, Xiang Li, Jiacheng Chuan, Nemanja Kuzmanović, Marie-Agnès Jacques, Perrine Portier, Ebrahim Osdaghi

**Affiliations:** 1 Department of Plant Protection, School of Agriculture, Shiraz University 37551, Shiraz, Iran; 2 Department of Plant Protection, College of Agriculture, University of Tehran 734129, Karaj, Iran; 3 Department of Plant Protection, College of Agriculture, Isfahan University of Technology 48456, Isfahan, Iran; 4 Plant Pathology & Environmental Microbiology Department, The Pennsylvania State University 8082, University Park, Pennsylvania, USA; 5 Canadian Food Inspection Agency (CFIA), Charlottetown Laboratory, Charlottetown, Canada; 6 Julius Kühn-Institut (JKI), Federal Research Centre for Cultivated Plants, Institute for Plant Protection in Horticulture and Urban Green 98882, Braunschweig, Germany; 7 Univ Angers, Institut Agro, INRAE, IRHS, SFR QUASAV, CIRM-CFBP 628037, Angers, France; The University of Tennessee Knoxville, Knoxville, Tennessee, USA

**Keywords:** barley, grasses, *Hordeum vulgare*, Iranian Plateau, *Poaceae*, small grain cereals, *Triticum* spp.

## Abstract

Microbial plant pathogens possess higher diversity in the center of domestication of their host plants than in the introduced geographic area of the corresponding crops. In this study, the center of wheat, barley, and oat domestication in the Iranian Plateau was surveyed to shed light on the population structure, taxonomic diversity, and biology of *Clavibacter* species associated with these crops. Comprehensive field surveys completed with pathological assays and molecular-phylogenetic analyses showed that phylogenetically diverse groups of *Clavibacter* strains were associated with bacterial mosaic symptoms on small grain cereals in this domestication center. Besides the two previously described species, *Clavibacter tessellarius* and *Clavibacter zhangzhiyongii*, three clades of atypical *Clavibacter* strains, phylogenetically distinct from all described *Clavibacter* species, were isolated from wheat, barley, and oat in this study. Thus, *Clavibacter* strains associated with small grain cereals in the center of domestication of these crops belong to at least five taxonomically distinct clades, whereas simultaneous occurrence of multitaxa *Clavibacter* strains on small grain cereals has never been reported outside the Iranian Plateau. All five phylogenetic *Clavibacter* clades were capable of inducing mosaic and chlorosis on their host of isolation. Whole genome sequence-based comparative investigations showed that most virulence-associated genomic contents of the five *Clavibacter* clades isolated in this study were somewhat similar. Data obtained in this study suggest a correlation between the center of wheat, barley, and oat domestication and the high taxonomic diversity of the accompanying *Clavibacter* pathogens. Additional phylogeographic investigations in the area would strengthen this co-evolutionary hypothesis.

**IMPORTANCE:**

Members of the gram-positive corynebacterial genus *Clavibacter* sp. (family *Microbacteriaceae*) are seed-borne plant pathogens causing economically important plant diseases on annual crops and vegetables. While comprehensive global-scale studies have been conducted to study the population structure of *Clavibacter* species infecting tomato, potato, and pepper, phylogenomics investigations have not yet been conducted to monitor the taxonomic diversity, pathogenicity, and origin of *Clavibacter* strains pathogenic to small grain cereals. Archaeobotanical investigations suggested that human agriculture was established ≈11,000 years ago in the foothills of the Zagros Mountains in Northwestern Iran, while wheat and barley were among the very first crops domesticated in these areas. Thus, the analyses of taxonomic diversity and population structure of *Clavibacter* strains in the Iranian Plateau would shed light on the correlation between the center of domestication of these crops and the center of diversity of accompanying bacterial pathogens. Our findings showed that most of the *Clavibacter* strains isolated from small grain cereals in Iran belonged to the two previously described species *Clavibacter tessellarius* and *Clavibacter zhangzhiyongii*, while several strains were grouped in three distinct clades, all representing hypothetical novel species in the genus.

## INTRODUCTION

Human agriculture was established ≈11,000 years ago in the foothills of the Zagros Mountains, somewhat between Northwestern Iran, Southwestern Turkey, and Northeastern Iraq (1). This area is often known as the Fertile Crescent and is considered the cradle of human civilization. Small grain cereals, such as wheat and barley, were among the very first field crops domesticated in the Fertile Crescent. According to the archaeobotanical investigations, einkorn wheat (*Triticum monococcum*) was the first wheat species that humans domesticated around 10,000 years ago, initiating the Neolithic revolution (2). Emmer wheat (*Triticum turgidum*) was also domesticated west of the Euphrates (Iraq), from where it gradually moved to Southeast Turkey (3). Archeological investigations of early agricultural settlements showed that wild einkorn wheat lines from the Karacadağ Mountains (Southeast Turkey) are the ancestors of cultivated einkorn varieties (4). Furthermore, archeological sites in Northeastern Iran, such as Sang-i-Chakhmaq, included various wheat species, namely emmer and einkorn. These crops spread eastward across the Iranian Plateau, eventually reaching India and China (5). Archeological investigations on the remains of barley (*Hordeum vulgare*) grains revealed that the crop was domesticated ≈10,000 years ago in the Fertile Crescent (6). Zhou et al. (7) also found a close association between several oat (*Avena sterilis*) accessions from the Iran-Iraq-Turkey region and cultivated accessions, suggesting that all cultivated hexaploid oat species are derived from progenitor germplasm from Southwest Asia, present-day Iran, Iraq, and Turkey. Development of early stock rearing has also been confirmed in the same region (4, 8, 9). Thus, the Iranian wheat, barley, and oat growing areas, especially those located in the western and northwestern provinces of the country, are considered the center of domestication of these crops (1).

Domestication of agricultural crops, which led to their extensive cultivation, correlated with the emergence and evolution of concomitant microbial pathogens. For most cultivated plant species, the center of domestication of an agricultural crop is considered the center of diversity of their corresponding pathogens. On the other hand, a number of plant pathogens have been first described in the introduced geographic range, somewhere outside the center of origin of their host plant (10). Knowing the center of genetic diversity of microbial plant pathogens and the pathways of geographic spread of a pathogen is of pivotal importance. This is helpful to combat further spread of the pathogen and identify its natural enemies. As far as small grain cereals are concerned, the Iranian Plateau is considered the center of diversity of most of the pathogens infecting these crops. For instance, wheat and the fungal wheat pathogen, *Zymoseptoria tritici,* share their center of diversity in Northwestern Iran (11). Emergence and evolution of the barley-infecting fungal pathogen *Zymoseptoria passerinii* has also been documented in the same area (12). Furthermore, the Iranian Plateau is considered the center of diversity of the bacterial wheat pathogen *Xanthomonas translucens* (13). However, some of the wheat and barley pathogens were initially described in an area outside the Iranian Plateau, even in the New World, challenging the above-mentioned co-evolutionary theory.

The gram-positive bacterium *Clavibacter tessellarius* was first isolated from winter wheat plants in Nebraska (USA) in 1976, causing bacterial mosaic disease (14). The species is a member of the *Microbacteriaceae* family, commonly known as coryneform bacteria or corynebacteria (15). For three decades after its description, *C. tessellarius* has been thought to be restricted to North America. There was no trace of the pathogen in the Old World until recently, when *C. tessellarius* was isolated from wheat plants in Iran (16, 17). Another species, phylogenetically closely related to *C. tessellarius,* causing a similar disease, has recently been isolated from barley and described as *Clavibacter zhangzhiyongii* in China. Indeed, the two *C. zhangzhiyongii* strains, DM1^T^ and DM3, were originally isolated from barley seeds imported from Australia to China, geographically far from the center of domestication of the host plant (18). These findings were not in congruence with the above-mentioned theory, which anticipates a shared center of diversity for the microbial pathogens and their host plants. This incongruence led us to question whether the center of diversity of *Clavibacter* species infecting small grain cereals is different from the center of domestication of their host plants, such as wheat, barley, and oat. This study aims to address the question via comprehensive field surveys and samplings throughout small grain cereals (i.e*.,* wheat, barley, and oat) producing areas in Iran and the investigation of *Clavibacter* strains isolated from plant specimens. Molecular phylogenetic analyses suggested that the center of wheat domestication drives the diversity of the *Clavibacter* pathogen since several previously unnoticed lineages of the bacterium are associated with small grain cereals in the Iranian Plateau.

## RESULTS

### Surveys, sampling, and bacterial strains

**Fig 1:**
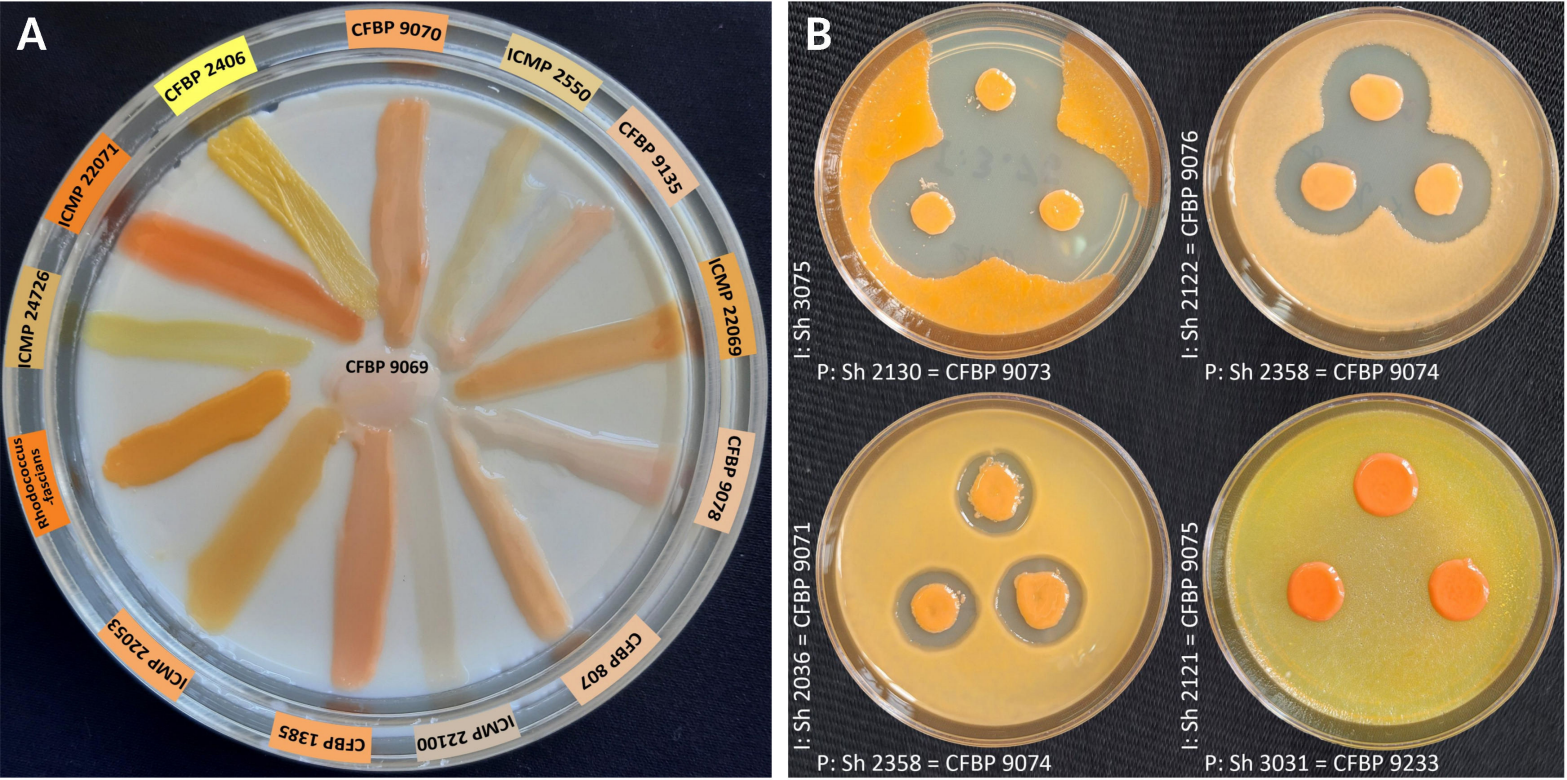
(**A**) Colony color and pigmentation of *Clavibacter* strains isolated in this study compared with other corynebacterial plant pathogens on YDC medium. (**B**) Bacteriocin production by *Clavibacter* strains obtained in this study, and those of different plant pathogenic corynebacteria (Fig. S1). Strains spotted before the challenge were potential producers, whereas the strains spread over the surface of the YPGA plates were indicator strains. Clear zones appeared around the colonies of producer strains, indicating inhibition of indicator strains in 48 h. The plates were arranged right-left from the highest inhibitory to no inhibitory. I: indicator, P: producer.

During 2020–2022, field surveys and samplings were conducted in small grain cereals-producing areas in Iran to monitor the status of *Clavibacter* infection on these crops. Samplings were focused on western and northwestern provinces of the country that are considered the center of domestication of wheat. Besides cultivated wheat, barley (*Hordeum vulgare*), and oat (*Avena sativa*) plants were also evaluated for infection with *Clavibacter* spp. Dozens of gram-positive bacterial strains resembling the members of the coryneform bacteria were isolated from plant specimens. All bacterial strains were subjected to morphological, biochemical, and DNA sequence-based molecular investigations as recommended for the *Clavibacter* species. All strains that were suspected to be members of *Clavibacter* possessed gram-positive non-motile cells with orange-to-peach-pigmented colonies on general culture media, as shown in Fig. 1A and B. Colonies were circular (2–4 mm in diameter), convex, and glistening and had entire margins. Preliminary phenotypic investigations accomplished with *Clavibacter*-specific PCR primers suggested that among the bacterial strains isolated in this study, 25 strains were members of *Clavibacter*. The remaining strains were assigned to either *Curtobacterium* (27 strains), *Microbacterium,* or other genera within the *Microbacteriaceae* family. Details of non-*Clavibacter* strains isolated in this study were described elsewhere (19). The 25 *Clavibacter* strains were isolated throughout central, western, northwestern, and southern Iran. Nineteen of 25 strains were isolated from wheat, whereas three strains were isolated from each of barley and oat plants (Table 1).

**TABLE 1:** Bacterial strains obtained in this study, their taxonomy, origin, and GenBank accession numbers^a^

Strain	Other code	Identified as	Host	Origin		Year of isolation	GenBank accession no.
				Province	County		
Zarg2		*Clavibacter tessellarius* ^*b*^	Wheat	Fars	Zarghan	2020	NA
Fir2		*Clavibacter tessellarius* ^*b*^	Wheat	Fars	Firuzabad	2020	NA
Fas1		*Clavibacter tessellarius* ^*b*^	Wheat	Fars	Fasa	2020	NA
Sarv3		*Clavibacter tessellarius* ^*b*^	Wheat	Fars	Sarvestan	2020	NA
Sir2		*Clavibacter tessellarius* ^*b*^	Wheat	Kerman	Sirjan	2020	NA
Raf2		*Clavibacter tessellarius* ^*b*^	Wheat	Kerman	Rafsanjan	2020	NA
Ram1		*Clavibacter tessellarius* ^*b*^	Wheat	Khuzestan	Ramhormoz	2020	NA
Ahv1		*Clavibacter tessellarius* ^*b*^	Wheat	Khuzestan	Ahvaz	2020	NA
Dez1		*Clavibacter tessellarius* ^*b*^	Wheat	Khuzestan	Dezful	2020	NA
Bor1		*Clavibacter tessellarius* ^*b*^	Wheat	Bushehr	Borazjan	2020	NA
Sh2113	CFBP 9248	*Clavibacter tessellarius*	Wheat	Zanjan	Zanjan	2021	NA
Sh2121	CFBP 9075	*Clavibacter tessellarius*	Wheat	Qazvin	Qazvin	2021	JBLJAQ000000000
Sh2122	CFBP 9076	*Clavibacter tessellarius*	Wheat	Qazvin	Qazvin	2021	JBLJAR000000000
Sh3031	CFBP 9233	*Clavibacter tessellarius*	Barley	Ilam	Eyvan	2022	NA
Sh3086	CFBP 9398	*Clavibacter tessellarius*	Wheat	Zanjan	Zanjan	2022	NA
Sh3075		*Clavibacter tessellarius*	Wheat	Fars	Bajgah	2022	NA
Sh2130	CFBP 9073	*Clavibacter zhangzhiyongii*	Barley	Qazvin	Kamal Abad	2021	JBLJAO000000000
Sh2358	CFBP 9074	*Clavibacter zhangzhiyongii*	Oat	East Azerbaijan	Marand	2021	JBLJAP000000000
Sh3003	CFBP 9135	*Clavibacter zhangzhiyongii*	Barley	Golestan	Gonbad-e Kavus	2022	JBLJAL000000000
Sh2141	CFBP 9069	*Clavibacter* sp.	Wheat	Alborz	Karaj	2021	JBLFDT000000000
Sh3038	CFBP 9450	*Clavibacter* sp.	Wheat	Ilam	Eyvan	2022	NA
Sh2088	CFBP 9070	*Clavibacter* sp.	Wheat	East Azerbaijan	Marand	2021	JBLJAM000000000
Sh3027	CFBP 9243	*Clavibacter* sp.	Wheat	Kohgiluyeh-Boyer-Ahmad	Yasuj	2022	NA
Sh2036	CFBP 9071	*Clavibacter* sp.	Oat	Fars	Bajgah	2021	JBLJAN000000000
Sh2126	CFBP 9077	*Clavibacter* sp.	Oat	Zanjan	Zanjan	2022	JBLJAS000000000

^*a*^ NA, not available.

^*b*^ These strains were isolated by Nasiri and colleagues (17).

### Plant symptoms and pathogenicity assays

**Fig 2:**
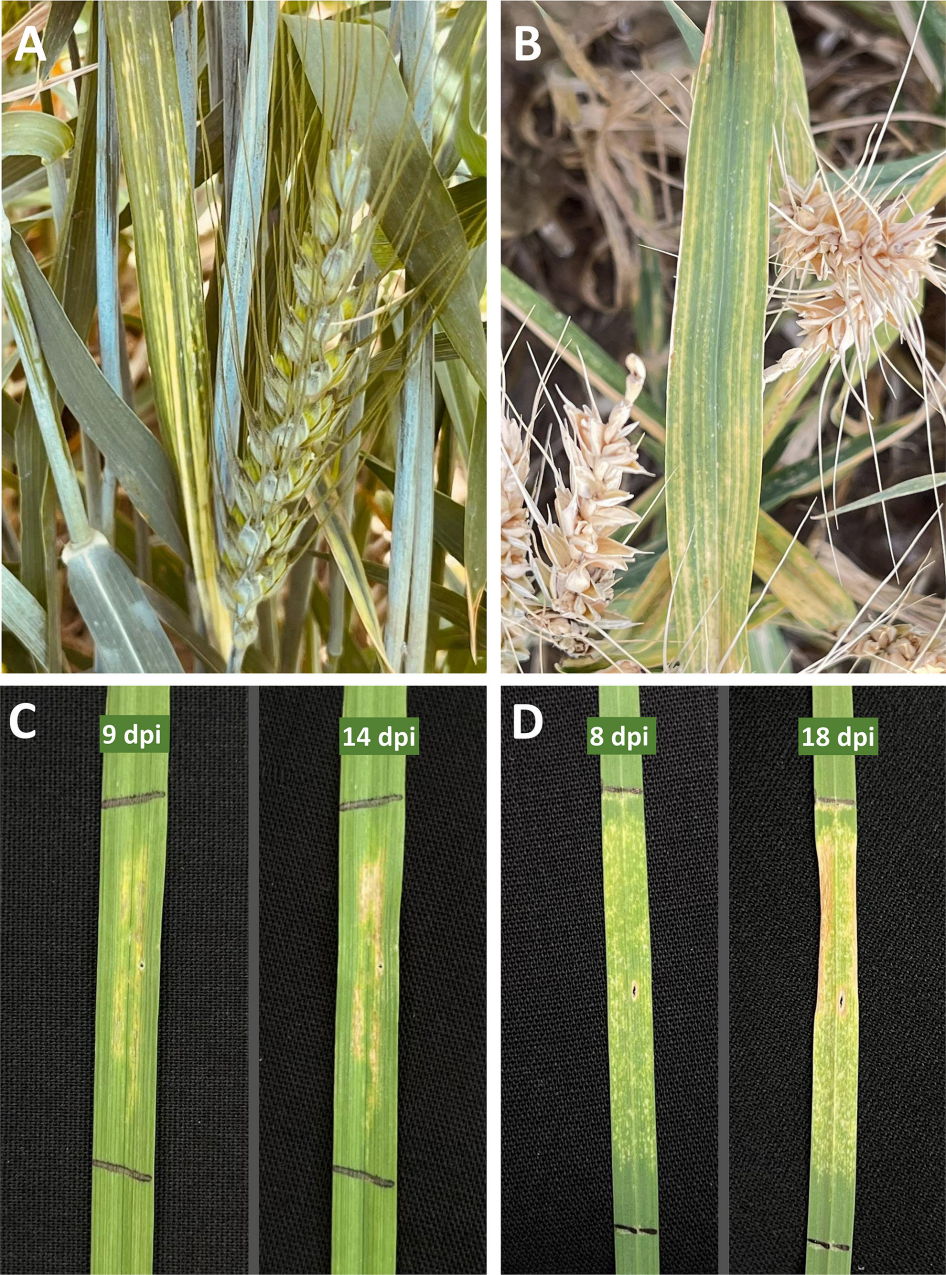
Field symptoms of bacterial mosaic of wheat caused by *Clavibacter* spp. (**A and B**). The symptoms included small yellow lesions with indefinite margins throughout the leaf, giving a mosaic appearance to the symptomatic tissues. Artificial inoculation of the strains on wheat (**C**) and barley (**D**) plants under greenhouse conditions led to the development of symptoms 3–5 days post-inoculation, resembling those observed under natural conditions.

Small grain cereals from which the bacterial strains were isolated showed yellow lesions on leaves with indefinite margins, giving a mosaic appearance to the infected tissues. Small lesions on leaves sometimes merged to form streaks. Symptoms were generally observed in the upper leaves of the plants, called the flag leaf (Fig. 2A and B). In some cases, the symptoms resembled nutrient deficits, such as iron deficiency, in wheat. Severely infected leaves turned brown and desiccated. Neither oozing nor water-soaking was observed on the infected plant tissues. The latter characteristics were differentiating features between the *Clavibacter* infection and bacterial leaf streak symptoms caused by *Xanthomonas translucens*.

To assess the pathogenicity of the bacterial strains on the host plants under greenhouse conditions, all 25 strains were inoculated on wheat, barley, and oat plants. All evaluated strains were capable of inducing symptoms on their host of isolation, as well as a few other grass species (Fig. 2C and D). The symptoms observed under artificial inoculation conditions included slight discoloration, yellowing, and tissue necrosis on the leaves. In some cases, gradual desiccation of the inoculated areas was also observed. In artificial inoculation under greenhouse conditions, initial symptoms were observed within 3–5 days post-inoculation. The pathogen did not spread to uninoculated tissues of the same plant nor to the vicinity plants (Fig. 2C and D).

### Molecular phylogenetic analyses

**Fig 3:**
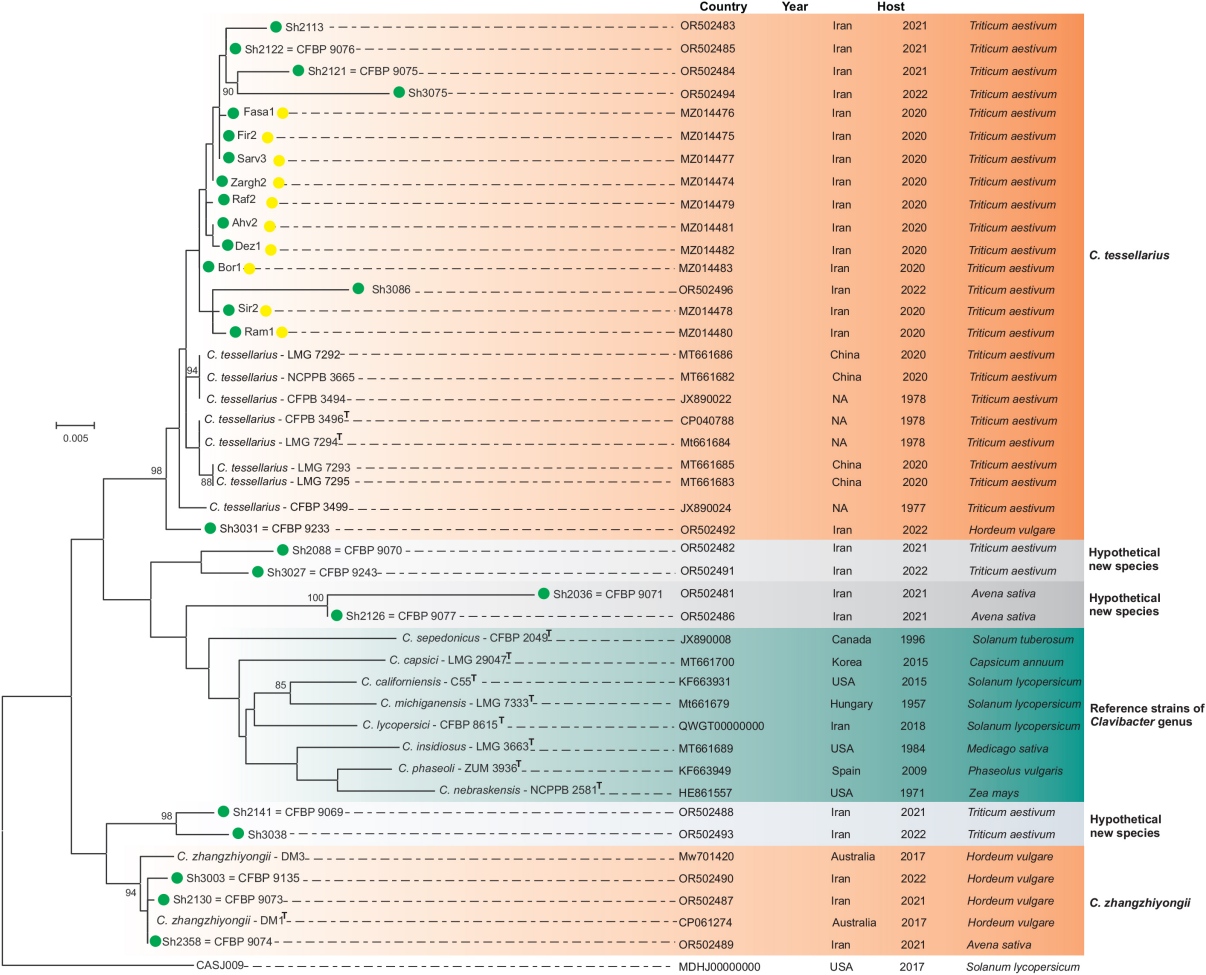
Phylogeny of *Clavibacter* strains isolated in this study based on the concatenated sequences of four housekeeping genes, that is, *atpD, gyrB, ppk*, and *recA*. The phylogenetic tree was constructed with MEGA 7.0 software, based on maximum likelihood and using the general time reversible (GTR) model. Green circles indicate bacterial strains isolated from small grain cereal plants in this study. Most *Clavibacter* strains isolated in this study belonged to *C. tessellarius*, three strains were identified as *C. zhangzhiyongii*, and six strains, Sh2036, Sh2126, Sh2088, Sh3027, Sh2141, and Sh3038, clustered separately from all 10 validly described *Clavibacter* species. T: type strain. The bootstrap values < 80% are hidden in the phylogenetic tree. The strains marked with a yellow circle (10 strains) were adopted from the previous work by Nasiri et al. (17).

*Clavibacter* strains isolated from small grain cereals were subjected to molecular phylogenetic analyses using both multi-locus DNA sequencing (MLSA) (i.e*., atpD*, *gyrB*, *ppk*, and *recA* genes) of all strains and whole genome sequencing of representative strains. The results of multi-locus sequence analyses showed that out of 25 *Clavibacter* strains, 16 strains were clustered within the *C. tessellarius* clade (Fig. 3). Among the 16 *C*. *tessellarius* strains, 15 strains were isolated from wheat, whereas the strain Sh3031 was isolated from barley. The barley strain Sh3031 was clustered within the *C. tessellarius* clade closely to, but still distinct from, the wheat strains as shown in Fig. 3.

The strains Sh3003 and Sh2130, isolated from barley in Golestan and Qazvin Provinces, respectively, and Sh2358, isolated from oat in East Azerbaijan Province, clustered in the *C. zhangzhiyongii* clade. Two strains, Sh2141 and Sh3038, isolated from wheat in Alborz and Ilam Provinces, respectively, clustered together, phylogenetically closely related to *C. zhangzhiyongii,* but still distinct from the latter species as well as all previously described *Clavibacter* species (Fig. 3). The strains Sh2036 and Sh2126, isolated from oat in Fars and Zanjan Provinces, respectively, clustered together in a unique group apart from *C. tesselarius* and *C. zhangzhiyongii*. Furthermore, two strains, Sh2088 and Sh3027, isolated from wheat in East Azerbaijan and Kohgiluyeh-Boyer-Ahmad, respectively, were phylogenetically distinct from all *Clavibacter* species. Since MLSA revealed three novel phylogenetic clades within the *Clavibacter* strains isolated in this study, further investigations were implemented using whole genome sequencing of nine representative strains to clarify the exact taxonomic position of the above-mentioned atypical strains. Selection of the strains for whole genome sequencing was based on the phylogenetic position, host of isolation, and phenotypic features of the strains.

**Fig 4:**
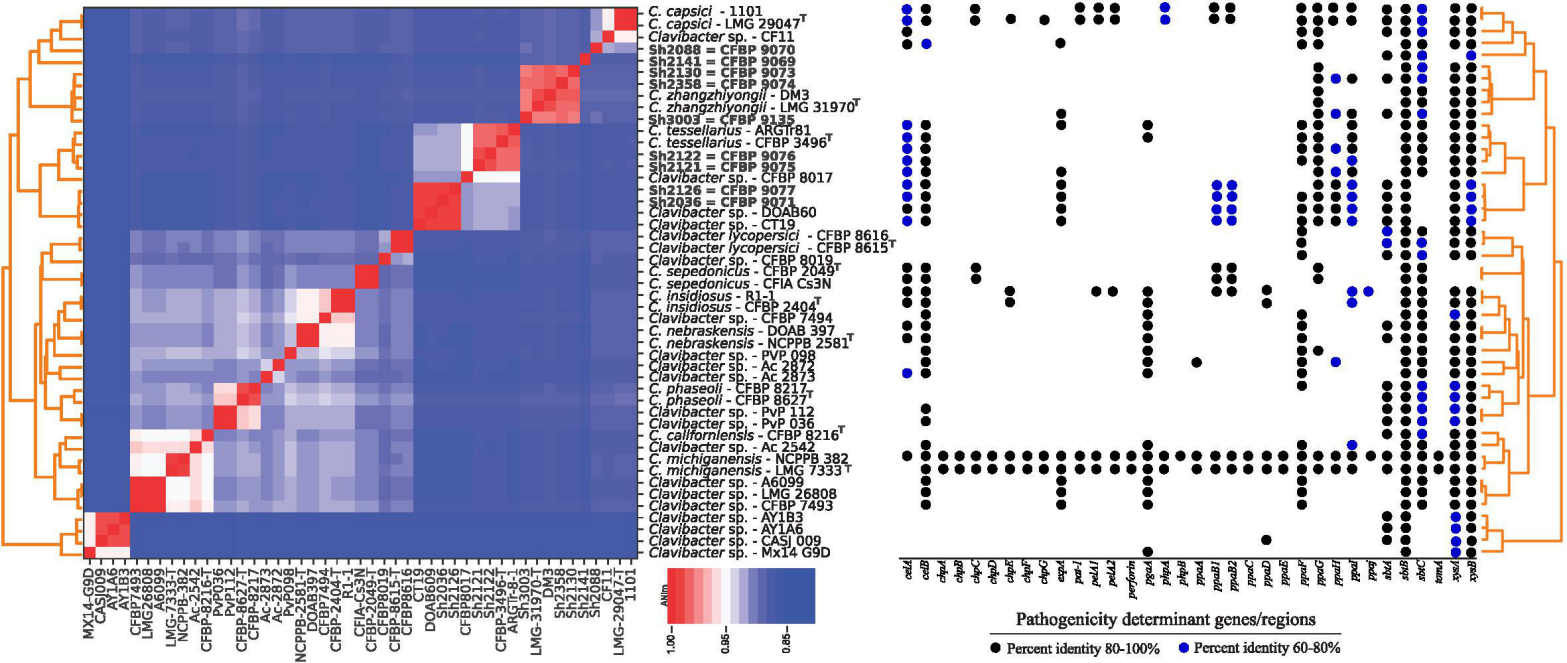
Average nucleotide identity-based pyANI matrix (right side) demonstrating nucleotide-level genomic similarity between the *Clavibacter* strains isolated in this study and type strains of all validly described species in the genus. The results of one-vs-one BLAST searches using the sequences of pathogenicity determinant genes/regions in the genome of *Clavibacter michiganensis* NCPPB 382 (GenBank: AM711867.1) against the strains sequenced in this study (left side).

BLASTn-based average nucleotide identity (ANIb) and digital DNA-DNA hybridization (dDDH) indexes were calculated using whole genome sequences of representative members of the above-mentioned atypical clades obtained in this study (Table 1), along with all validly described *Clavibacter* species. The results showed that the bacterial strains isolated from small grain cereals in this study could be assigned into five standalone *Clavibacter* species. Among these five species, *C. tessellarius* and *C. zhangzhiyongii* have already been validly described in the literature, whereas three novel phylogenetic clades were hypothetical novel species within the genus, as shown in Fig. 4. Table S1 includes detailed ANIb and dDDH indices among the authentic *Clavibacter* species and those of hypothetically novel species isolated in this study.

The two previously described cereal pathogens, *C. tessellarius* and *C. zhangzhiyongii,* are not closely related to each other based on their DNA similarity indices. According to the ANI matrix, the strains identified as *C. zhangzhiyongii* were phylogenetically closely related to the pepper pathogen *Clavibacter capsici,* as well as the two atypical strains Sh2088 and Sh2141. The *C. tessellarius* strains, however, were clustered distinct from the *C. zhangzhiyongii* clade, closely related to the strains Sh2126 and Sh2036. The strain Sh2088 showed ANI < 92.5% and dDDH <49% with type strains of all *Clavibacter* species, which was far below the accepted threshold for species definition in prokaryotes (ANI > 95% and dDDH >70%). Thus, the strains Sh2088 and Sh3027 should be assigned to a standalone species as shown in Fig. 3. Furthermore, the strains Sh2036 and Sh2126 showed 99.2% ANI and 93.4% dDDH with each other, while they differed from all type strains of the *Clavibacter* species with ANI < 93.1 and dDDH <50% as detailed in Table S1. The latter two strains could also be assigned to a novel species within the genus. The strain Sh2141 also showed ANI < 91% and dDDH <41% with type strains of *Clavibacter* spp., indicating its distinct taxonomic position from the previously described species. Thus, the strains Sh2141 and Sh3038 could be considered a novel standalone species of *Clavibacter* as shown in Fig. 3.

High taxonomic diversity among the *Clavibacter* strains isolated from small grain cereals in Iran led us to speculate whether the genomic content and arrangement of pathogenicity determinant elements varied among those strains. Thus, a one-vs-one BLAST-based search was conducted to find the genomic elements with significant contribution to the pathogenicity of *Clavibacter* species in the whole genome of the strains obtained in this study. No significant variation was observed among different small-grain cereals-associated *Clavibacter* species in their pathogenicity-related genes. The only noticeable difference between the strains was *expA*, *pgaA*, and *sbtA* genes, which varied among phylogenetic clades (Fig. 4).

### Antibiosis

Antibiosis and biological competition among the strains isolated in this study were evaluated using a plate-based dual-culture method. Type strains of *Clavibacter* species and reference strains of other plant-associated coryneform bacteria were also included in the antibiosis test. The strains with antibiosis activity inhibited the growth of their accompanying strain on YPGA medium, as shown in Fig. 1B. Three *C. zhangzhiyongii* strains had the highest inhibitory effect on the coryneform bacterial species, followed by *C. tessellarius,* as detailed in Fig. S1. The strains Fas2 and Sh3031, identified as *C. tessellarius* and the *Clavibacter* strain Sh3027, showed the least susceptibility to the inhibitory compounds produced by the encountered strains. On the other hand, the type strain of *Rathayibacter tritici* CFBP 1385^T^ was the most susceptible strain against the antimicrobial compounds produced by the bacterial strains. Hence, *C. zhangzhiyongii* and *C. tessellarius* could potentially outcompete other *Clavibacter* strains isolated from small grain cereals as well as other plant pathogenic corynebacteria. Specifically, *C. zhangzhiyongii* was consistently aggressive compared with the other strains used in this study.

## DISCUSSION

In this study, we assessed geographic distribution, pathogenicity, and taxonomic diversity of *Clavibacter* spp. strains associated with symptomatic small grain cereals in Iran. Extensive surveys were conducted in major small grain cereal-growing areas across 15 provinces. Different symptoms, including mosaic, leaf chlorosis, and necrotic lesions, were observed on wheat, barley, and oat plants. The results of the phylogenetic analyses showed that most *Clavibacter* strains belonged to *C. tessellarius*, whereas three strains were identified as *C. zhangzhiyongii*, and six strains, Sh2036, Sh2126, Sh2088, Sh3027, Sh2141, and Sh3038, clustered separately from all 10 validly described *Clavibacter* species. The latter six strains were grouped into three distinct clades, all hypothetically representing novel species in the genus.

The genus *Clavibacter* was first described in 1984, including several distinct members pathogenic on alfalfa, maize, pepper, potato, tomato, and wheat (20). Until 2014, the genus included only one species, namely *C. michiganensis sensu lato,* with five subspecies (21, 22). During 2014–2016, additional members—either pathogenic or non-pathogenic—have been described as novel subspecies (23–25). By 2016, *C. michiganensis* included eight subspecies, six of which were pathogenic on their host of isolation. In 2018, reclassification of *C. michiganensis* with elevation of all subspecies at the species level was proposed (26). Lately, two new species have also been described: *Clavibacter zhangzhiyongii,* the causal agent of barley brown leaf spot (18), and *Clavibacter lycopersici,* a non-pathogenic bacterium associated with symptomless tomato plants (27). Thus, *Clavibacter* currently comprises 10 validly described standalone species*—Clavibacter californiensis* (isolated from tomato), *Clavibacter capsica* (pepper), *Clavibacter insidiosus* (alfalfa), *Clavibacter lycopersici* (tomato), *Clavibacter michiganensis sensu stricto* (tomato), *Clavibacter nebraskensis* (maize), *Clavibacter phaseoli* (common bean and tomato), *Clavibacter sepedonicus* (potato)*, Clavibacter tessellarius* (wheat), and *Clavibacter zhangzhiyongii* isolated from barley (28–30).

A correlation is conceivable between the high taxonomic diversity of *Clavibacter* pathogens and the center of domestication of the corresponding host plants. For instance, both maize and tomato are domesticated in the New World, where *C. nebraskensis*, *C. michiganensis*, *C. californiensis*, and *C. phaseoli* (formerly *Clavibacter michiganensis* subsp. *chilensis*) were originally described (27). Seven out of 10 *Clavibacter* species were originally described in the New World, *C. sepedonicus* was described in Europe, whereas *Clavibacter capsica*, *C. lycopersici*, and *C. zhangzhiyongii* were described in Asia (18). Considering the center of domestication of the corresponding host plants, tomato, potato, pepper, and maize originated from America, whereas alfalfa, barley, and wheat originated from the Middle East and the Fertile Crescent (1). Interestingly, six of 10 *Clavibacter* species are associated with members of the *Solanaceae* family, such as tomato, potato, and pepper (31), although they vary in the host range and aggressiveness on different nightshade plants (32, 33). Thus, one could assume that *Clavibacter* members are tightly associated with nightshade plants regardless of the pathogenicity status of the strains on the host plants. Our data, however, would change the taxonomic framework of *Clavibacter* species with the introduction of three novel phylogenetic clades isolated from small grain cereals in Iran. When validly described, these three clades will represent three novel species in the genus, changing the ratio of species associated with grasses (*Poaceae* family) compared with the number of species isolated from other plant species. Ultimately, *Clavibacter* species associated with small grain cereals will be taxonomically scattered within five standalone species, that is, *C. tessellarius*, *C. zhangzhiyongii*, and three hypothetically novel species isolated in this study.

Concerning *Clavibacter* members pathogenic to small grain cereals, *C. tessellarius* was originally described in the USA, while *C. zhangzhiyongii* was described in China from barley seeds imported from Australia (14, 18). None of these areas is considered the center of domestication of the corresponding host plants (1). We have isolated both *C. tessellarius* and *C. zhangzhiyongii* from small grain cereals in this study, in addition to three hypothetically novel species. A similar correlation is observed in the other corynebacterial plant pathogen, *Curtobacterium flaccumfaciens,* and common bean (*Phaseolus vulgaris*) as the host plant (34, 35). The center of domestication of the common bean and the center of diversity of *Curtobacterium flaccumfaciens* are based in the American continent (36). Furthermore, the central highlands of Mexico are considered the center of domestication and diversity for both potato and its late blight pathogen *Phytophthora infestans* (37, 38). Asian cultivated rice (*Oryza sativa*) was first domesticated in East Asia, whereas African rice (*Oryza glaberrima*) has origins in Africa (39). Parallel to the host plant, rice-pathogenic bacterium *Xanthomonas oryzae,* causing bacterial blight and leaf streak disease, has two centers of diversity in East Asia and Africa (40, 41). Southeast Asia is also the center of origin, diversity, and dispersion of the rice blast fungus *Magnaporthe oryzae* (42).

The major issue that remains to be addressed is why *C. tessellarius* has originally been described in the USA several decades before its description in the center of domestication of the host plant. The question could be answered considering the following two points. First, plant bacteriology studies have been started in Iran far later than those implemented in the USA. Second, small-grain cereals-associated *Clavibacter* pathogens are relatively slow-growing microorganisms demanding enriched semi-selective culture media for *in vitro* growth. These bacteria could be easily outcompeted on culture media by saprophytic microorganisms or other pathogenic bacteria, such as *Xanthomonas translucens* (43, 44). Thus, for successful isolation and identification of these bacteria, an experienced person, set-up laboratory conditions, and specific culture media are required. During the 1970s and 1980s, all these prerequisites were available in the Department of Plant Pathology at the University of Nebraska-Lincoln, where Prof. Anne K. Vidaver was leading a world-class research program on plant pathogenic corynebacteria (45).

It could be hypothesized that *C. tessellarius* has been transmitted from the Iranian Plateau into the New World via infected wheat seeds. Since *C. tessellarius* is the only species of the genus isolated from small grain cereals in the USA, it seems that only a fraction of the taxonomic diversity and natural population of the pathogen has been transmitted to the New World, and a population expansion has occurred in the USA after a recent bottleneck. Additional phylogeographic investigations are needed to prove these speculations. Similar findings were reported for the bacterial leaf streak pathogen *X. translucens* pv. *undulosa,* which was described for the first time in the early 20th century in the USA (46). However, the bacterium was reported in Iran in the 1980s, based on molecular-phylogenetic investigations. The Iranian Plateau is considered the center of diversity of the pathogen (13). The pathotype strain of *X. translucens* pv. *undulosa* LMG 892^PT^ was isolated from a *Triticum turgidum* cultivar known as “Khorasan wheat” in the USA (47). The name of the host plant refers to “Khorasan Province” in Northeastern Iran and indicates the origin of the crop in the Iranian Plateau in the ancient civilizations (47). Khorasan wheat was accidentally introduced into the USA in 1949 and planted for the first time in Fort Benton, Montana. Commercial cultivation of the crop started in 1964. Soon after, the pathotype strain of *X. translucens* pv. *undulosa* LMG 892^PT^ was isolated in Southern Canada in 1966 (47).

In conclusion, data obtained in this study propose a correlation between high diversity of *Clavibacter* pathogens infecting small grain cereals (i.e*.,* wheat, barley, and oat) and the center of domestication of those plants. Sporadic occurrence of *Clavibacter* species in North America and China is more likely due to transmission of the bacteria via infected wheat and barley seeds. Although a taxonomically complex *Clavibacter* population, including five standalone species, is associated with small grain cereals in the Iranian Plateau, only *C. tessellarius* and *C. zhangzhiyongii* were reported in the USA and China, respectively. Occurrence of the latter pathogens has not yet been recorded in South America, Europe, Africa, and Australasia (48). *Clavibacter* spp. are considered minor pathogens of cereals in both the center of domestication of the host plants and the introduced geographic range of those crops. However, knowing where these pathogens have evolved is of high importance in terms of both plant disease management and international quarantine affairs (49). In addition, the probability of finding natural enemies able to control a pathogen is higher in the center of diversity rather than in the introduced range of a pathogen. Furthermore, knowing the center of origin and diversity of a plant pathogen can help us to understand the pathways of geographic distribution or reduce repeated international introductions. With fewer introductions, the corresponding pathogen will have lower genetic diversity in the destination, with lower capability to overcome host resistance.

## MATERIALS AND METHODS

### Surveys, sampling, and bacterial isolation

During 2020–2022, comprehensive surveys and inspections were conducted in small grain cereal growing areas in Iran. Samplings included various provinces located in central (Alborz and Tehran), southern (Bushehr, Fars, Hormozgan, and Khuzestan), northwestern (East Azerbaijan, Qazvin, and Zanjan), northern (Golestan and Semnan), western (Ilam and Kurdistan), southwestern (Kohgiluyeh-Boyer-Ahmad), northeastern (Razavi Khorasan and South Khorasan), and southeastern (Sistan and Baluchestan) Iran. These locations are geographically close to the center of wheat domestication in the Zagros Mountains (1) and are considered the main areas for the cultivation of wheat, barley, and oat in the country. The sampling date varied within a cropping year in different areas due to different climatic conditions and agronomic practices, such as differences in the planting dates. The field surveys and samplings were comprehensive enough to cover both cultivated and wild grasses, including weed species. However, we picked only symptomatic grass specimens suspected to be infected with the bacterial mosaic agent. Cereal plants showing symptoms of suspected bacterial disease, such as bacterial mosaic or brown leaf spots, were collected from all surveyed regions and transported to the laboratory for additional examination. Isolation of the putative bacterial pathogens from plant specimens was carried out on yeast-extract peptone glucose agar (YPGA) medium using the procedure described by Schaad et al. (50). In brief, symptomatic pieces of each sample were cut using a sterile scalpel, surface sterilized by dipping into 0.5% sodium hypochlorite for 20s, followed by 2–3 rinses in sterile distilled water (SDW), and macerated in 10–20 mL of SDW. A loopful of the resulting suspension was streaked onto YPGA medium, the plates were incubated at 25–27°C for 72–96 h, and the resulting bacterial colonies were purified by repetitive streaking on fresh YPGA plates. For seed samples, approximately 50–100 seeds were randomly tested from each seed lot. The seeds were dipped into SDW for 30 s, rinsed three times, transferred into a sterile mortar containing 5 mL SDW, and ground with a sterile pestle. The resulting suspensions were used for bacterial isolation as described above. Purified bacterial strains were re-suspended in SDW and stored at 4°C for further use. Sterile 15% glycerol was used for their long-term storage at −70°C.

### Phenotypic characterization of the bacterial strains

All purified bacterial strains obtained in this study (Table 1) were subjected to standard biochemical tests. Gram reaction, aerobic/anaerobic growth (O/F), growth on 2,3,5-triphenyl tetrazolium chloride (TTC) medium, and colony characteristics on yeast extract-dextrose-calcium carbonate (YDC) agar medium were evaluated (50). Type strain of *Clavibacter tessellarius* CFBP 3496^T^ was included as a positive control. All biochemical tests were repeated twice.

### Pathogenicity assays

All bacterial strains were assessed for their pathogenicity on wheat (cv. Azadi), barley (cv. Rihane), and oat plants under greenhouse conditions using the procedure described previously (17, 51). The plants were grown in 20 cm diameter pots (five plants/pot), and they were maintained in a greenhouse at ambient conditions (25–27°C, 14 h natural light). Pathogenicity tests were conducted on 17-day-old plants when they had at least 3–4 expanded leaves. The bacterial suspension consisted of 1 × 10^8^ CFU/mL and was prepared from a 48 h old culture grown on YPGA medium. Bacterial suspensions were injected into the fully expanded leaves using a sterile insulin syringe, and the inoculated area was marked using a waterproof marker to ease the subsequent monitoring of the symptom development on the leaves. Four plants were inoculated with each of the bacterial strains described in Table 1. Inoculated plants were maintained in greenhouse conditions at 85%–90% relative humidity and monitored regularly for the appearance of disease symptoms until three weeks post-inoculation. All water soaking, discoloration, and mosaic symptoms were recorded in 3- to 4-day intervals. The same number of plants was inoculated with *C. tessellarius* (CFBP 3496^T^) and *C. michiganensis* (ICMP 2550^T^) as positive and negative controls, respectively. Koch’s postulates were fulfilled by re-isolation of inoculated bacterial strains from plants exhibiting disease symptoms on YPGA medium. The re-isolated bacterial strains were confirmed based on the morphology of the colonies (i.e*.,* colony pigment) on YDC medium, Gram test, as well as *Clavibacter*-specific PCR primers CMR16F1/CMR16R1 (52, Table S2). The pathogenicity tests were repeated three times.

### Molecular-phylogenetic analyses

The bacterial strains isolated from small grain cereals in this study were subjected to molecular-phylogenetic analyses to decipher their taxonomic status and phylogenetic relationships. All strains were tested using the primer pair CMR16F1/CMR16R1 specific for *Clavivacter* spp. (52). Type strains of *C. tessellarius* (CFBP 3496^T^) and *C. michiganensis* (ICMP 2550^T^) were included as controls. Culture preparation, bacterial DNA extraction, and PCR reaction methods were conducted using the procedure described previously (53). For PCR reactions, Universal PCR Kit, Ampliqon Taq DNA Polymerase Master Mix Red (Ampliqon A/S, Odense, Denmark) was used according to the manufacturer’s recommendations. For each strain, a 20 µL PCR including 50 ng total DNA and 1 µL of each primer (10 pmol/µL) was used. The annealing temperatures and the respective primer sequences are described in Table S2.

MLSA using the sequences of four housekeeping genes (i.e*., atpD*, *gyrB*, *ppk*, and *recA*) was performed to obtain an accurate and reliable taxonomic status and phylogenetic position of the strains isolated in Iran (21, 54). The annealing temperatures and the sequences of primer pairs are indicated in Table S2, whereas the PCR procedure was the same as described by Osdaghi et al. (53). The certified PCR products were sent to Bioneer Corporation (https://www.bioneer.com) for sequencing using Sanger sequencing technology. The resulting sequences were analyzed by BLASTn (http://blast.ncbi.nlm.nih.gov) and compared phylogenetically with those of the corresponding genes in a collection of *Clavibacter* strains, including 10 type strains of all *Clavibacter* species obtained from the GenBank database. All sequences were aligned using ClustalW (55) with MEGA 7.0 software (56) and concatenated in an alphabetic order, resulting in 1,686 bp of sequences, with nucleotides 1–471 for *atpD* (471  bp), 472–879 for *gyrB* (407  bp), 880–1,252 for *ppk* (372 bp), and 1,253–1,686 for *recA* (433 bp) genes. Phylogenetic trees were constructed via the maximum likelihood method with MEGA 7.0 software, using both single genes and concatenated sequences. The evolutionary model for maximum likelihood analysis was determined using the Modeltest tab in MEGA 7.0, and phylogenetic trees were constructed using the General Time Reversible model (57) with 1,000 bootstrap replicates.

Representative strains from different phylogenetic clades of *Clavibacter* spp. isolated in this study based on MLSA were subjected to whole genome sequencing to shed light on their phylogenetic positions, genomic features, and virulence repertoires (Table 1). Culture preparation, DNA extraction, and genome sequencing were the same as previously described (58). DNAs were sequenced via DNBseq and assembled using Unicycler v0.4.8. Genome annotation was performed using the GeneMarkS+ (v 4.6) suite implemented in the NCBI Prokaryotic Genome Annotation Pipeline with default settings. BLASTn-based average nucleotide identity (ANIb) and digital DNA-DNA hybridization (dDDH) indices were calculated among the strains sequenced in this study and all validly described *Clavibacter* species. ANI values were calculated using the following three different algorithms: JSpeciesWS, ANI calculator, and OrthoANIu (59, 60). Additionally, the Genome-to-Genome Distance Calculator online service (http://ggdc.dsmz.de/distcalc2.php) was used to calculate the digital DNA-DNA hybridization (dDDH) value, which refers to the genome-to-genome distances between pairs of genomes based on the Genome Blast Distance Phylogeny (61). For whole-genome sequence-based classification of the bacterial strains, the genome assemblies were used as input for the pyANI v0.2.11 Python pipeline (62), with the ANIm parameter to calculate pairwise distances using MUMmer (nucmer). The pipeline generates a distance matrix and a double hierarchical clustered heatmap, in which a red color indicates ANI percent identities above 95%.

In order to determine if genomic elements with significant contribution to pathogenicity of *Clavibacter* species were present in the genomes of the strains obtained in this study, sequences of these virulence genes, as summarized by Peritore-Galve et al. (63), were retrieved from the complete genome sequence of *C. michiganensis* NCPPB 382 (GenBank: AM711867.1). One-vs-one BLASTn/BLASTp search was implemented using the sequences of these genes (i.e*.,* a 129 kb low G + C pathogenicity island, which contains *chp* and *tomA* clusters; 63) against the whole genome sequences obtained in this study. Proteins with amino acid sequence similarities higher than 50% and query coverage higher than 70% were considered homologs. The complete genome sequence of *C. michiganensis* NCPPB 382 was used as a control.

### Antibiosis and bacteriocin production

The bacteriocin typing assay, based on the production of /or sensitivity to a certain bacteriocin, is useful to characterize biological features of corynebacterial plant pathogens (64, 65). Bacteriocins are antimicrobial compounds that are ribosomally synthesized and produced by bacteria to inhibit the growth of similar or closely related strains by various mechanisms (66). The bacteriocin-producing ability of all bacterial strains isolated in this study was assessed along with type strains of closely related plant pathogenic coryneform bacterial species. Type and/or reference strains of *Clavibacter* spp., *Curtobacterium* spp., *Rathayibacter* spp., and *Rhodococcus* sp. were included in this test. The experiments were conducted using the culture plate method originally described by Gross and Vidaver (65) with slight modifications as detailed previously (53). In brief, a bacterial suspension containing 1 × 10^5^ CFU/mL was prepared for all strains evaluated for antibiosis from the 24 h-old culture of the cells grown on YPGA medium. All bacterial strains were spotted on culture medium in three corners of an imaginary triangle and incubated at 27°C for 24 h. Bacterial strains that were cultured in these three spots were considered potentially capable of producing bacteriocins or other antibacterial compounds. In this assay, inhibitory compound(s) from producer strains diffuse into the surrounding medium and inhibit the growth of the indicator strains. The potential producer strains were grown for 24 hpi. Then, cell suspensions of the indicator strains were adjusted to 1 × 10^8^ CFU/mL. The indicator strains were paired with the producer strains in a one-to-all manner. Bacterial suspensions of the indicator strains were individually sprayed over the surface of the agar on which producer strains were grown using a spray atomizer. A clear zone of inhibition around the producer colonies 48 h post-spraying was considered indicative of antibiosis. The width of inhibition zones was measured as the distance between the edge of the producer culture and the outer edge of the inhibition zone on three replicate plates. SDW was used as a control. Conditional Formatting on the Home tab of the MS Excel 2016 ribbon was used to create the heatmap. The Red-Yellow-Green color scale was selected to display the color gradient in the range of the data cells. The experiment was conducted twice.

## Data Availability

The nucleotide sequences obtained in this study were deposited in the GenBank database under the following accession numbers: *atpD*, OR502450 to OR502464; *gyrB*, OR502481 to OR502496; *ppK*, OR502465 to OR502480; and *recA*, OR502497 to OR502511. Whole genome sequences of the representative strains were deposited in the GenBank database under the following accession numbers: JBLJAL000000000 for Sh3003, JBLJAM000000000 for Sh2088, JBLJAN000000000 for Sh2036, JBLJAO000000000 for Sh2130, JBLJAP000000000 for Sh2358, JBLJAQ000000000 for Sh2121, JBLJAR000000000 for Sh2122, JBLJAS000000000 for Sh2126, and JBLFDT000000000 for Sh2141. Further, a pure culture of the representative strains isolated in this study has been deposited in the CIRM-CFBP (https://cirm-cfbp.fr/) and ICMP (International Collection of Microorganisms from Plants Auckland, New Zealand) culture collections with the following accession numbers: Sh2141 = CFBP 9069 = ICMP 24735, Sh2088 = CFBP 9070 = ICMP 24732, Sh2036 = CFBP 9071 = ICMP 24729, Sh2130 = CFBP 9073 = ICMP 24733, Sh2358 = CFBP 9074 = ICMP 24731, Sh3003 = CFBP 9135 = ICMP 24734, Sh2121 = CFBP 9075, Sh2122 = CFBP 9076, and Sh2126 = CFBP 9077.

## SUPPLEMENTAL MATERIAL

Figure S1 aem.01245-25-s0001.pdf Bacteriocin production scheme of corynebacterial strains in this study.

Table S1 aem.01245-25-s0002.docx ANI and dDDH values among the type strains of *Clavibacter* species and the strains sequenced in this study.

Table S2 aem.01245-25-s0003.docx Primer pairs used in this study.

ASM does not own the copyrights to Supplemental Material that may be linked to, or accessed through, an article. The authors have granted ASM a non-exclusive, world-wide license to publish the Supplemental Material files. Please contact the corresponding author directly for reuse.
