# Supplementary material for: The center of wheat domestication drives diversity of Clavibacter pathogens
Source: Appl Environ Microbiol. 2025 Oct 8;91(11):e01245-25. doi: 10.1128/aem.01245-25 (PMC12628682; doi:10.1128/aem.01245-25)
Supplement: Figure S1 — Bacteriocin production scheme of corynebacterial strains in this study. [file aem.01245-25-s0001.pdf]

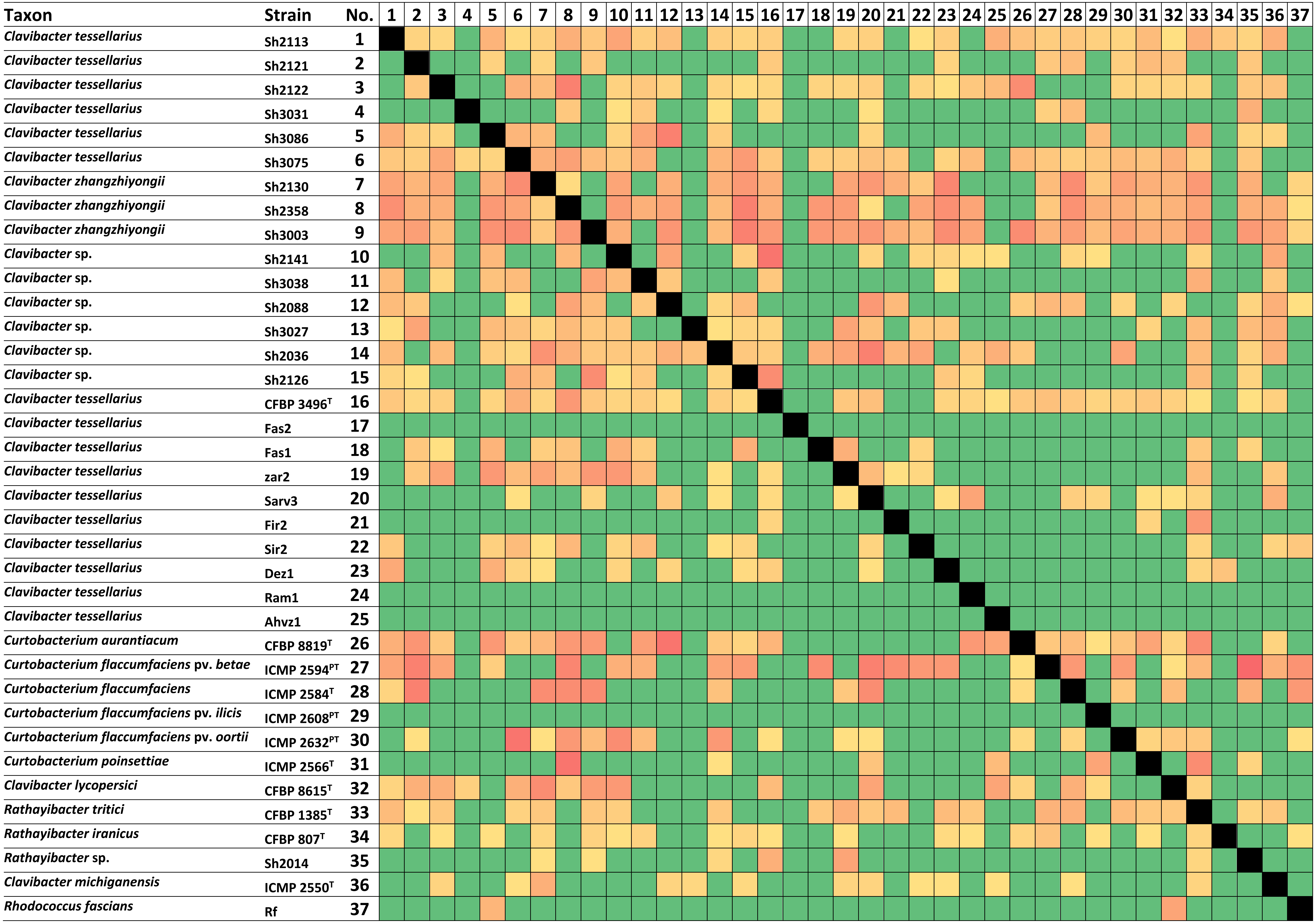

Figure S1: Bacteriocin production scheme of corynebacterial strains in this study. The leftmost column shows the producer strains, whereas the top row shows the indicator strains. The degree of inhibition is represented by Microsoft Excel® heat map capability, in which red cells indicate complete inhibition, whereas green cells indicate absence of visible inhibition.
