# Supplementary material for: The center of wheat domestication drives diversity of Clavibacter pathogens
Source: Appl Environ Microbiol. 2025 Oct 8;91(11):e01245-25. doi: 10.1128/aem.01245-25 (PMC12628682; doi:10.1128/aem.01245-25)
Supplement: Table S1 — ANI and dDDH values among the type strains of Clavibacter species and the strains sequenced in this study. [file aem.01245-25-s0002.docx]

**Table S1:** Average nucleotide identity (ANI; lower diagonal) and digital DNA–DNA hybridization (dDDH; upper diagonal) values among the type strains of *Clavibacter* species and the strains sequenced in this study (marked by *).

| **No.** | **Taxon** | **Strain** | **1** | **2** | **3** | **4** | **5** | **6** | **7** | **8** | **9** | **10** | **11** | **12** | **13** | **14** | **15** | **16** | **17** | **18** | **19** |
| --- | --- | --- | --- | --- | --- | --- | --- | --- | --- | --- | --- | --- | --- | --- | --- | --- | --- | --- | --- | --- | --- |
| **1** | ***Clavibacter* sp*.*** | **Sh2088*** |  | 37.90 | 37.90 | 38.10 | 38.10 | 41.00 | 41.10 | 43.80 | 39.80 | 40.00 | 39.40 | 39.20 | 40.50 | 41.00 | 43.0 | 41.40 | 47.80 | 38.20 | 41.30 |
| **2** | ***Clavibacter* sp*.*** | **Sh2036*** | 89.79 |  | 93.40 | 48.90 | 48.90 | 37.70 | 37.50 | 37.60 | 35.90 | 36.80 | 36.80 | 35.80 | 37.10 | 36.90 | 39.40 | 37.40 | 38.20 | 48.50 | 37.50 |
| **3** | ***Clavibacter* sp*.*** | **Sh2126*** | 89.78 | 99.24 |  | 48.90 | 48.80 | 37.70 | 37.50 | 37.60 | 35.90 | 36.70 | 36.70 | 35.90 | 37.00 | 36.90 | 39.30 | 37.50 | 38.20 | 48.40 | 37.50 |
| **4** | ***C. tessellarius*** | **Sh2121*** | 89.96 | 92.96 | 92.92 |  | 87.40 | 37.90 | 38.00 | 38.00 | 36.30 | 37.30 | 37.00 | 36.20 | 37.60 | 37.50 | 40.00 | 38.30 | 38.30 | 81.50 | 38.00 |
| **5** | ***C. tessellarius*** | **Sh2122*** | 89.92 | 93.01 | 92.90 | 98.68 |  | 37.80 | 37.90 | 38.00 | 36.30 | 37.20 | 37.00 | 36.10 | 37.60 | 37.50 | 39.80 | 38.20 | 38.10 | 81.40 | 38.10 |
| **6** | ***C. zhangzhiyongii*** | **Sh2130*** | 90.83 | 89.52 | 89.49 | 90.06 | 89.92 |  | 81.20 | 75.60 | 40.50 | 38.00 | 37.80 | 37.30 | 38.20 | 38.30 | 40.80 | 39.30 | 40.50 | 38.00 | 81.80 |
| **7** | ***C. zhangzhiyongii*** | **Sh2358*** | 90.82 | 89.52 | 89.44 | 90.03 | 89.97 | 97.87 |  | 81.30 | 40.10 | 37.70 | 37.50 | 37.20 | 38.10 | 38.30 | 40.50 | 39.00 | 40.40 | 37.90 | 82.00 |
| **8** | ***C. zhangzhiyongii*** | **Sh3003*** | 91.53 | 89.50 | 89.40 | 90.01 | 89.95 | 97.21 | 97.87 |  | 40.80 | 37.90 | 37.60 | 37.30 | 38.20 | 38.40 | 40.60 | 39.10 | 40.90 | 38.10 | 76.30 |
| **9** | ***Clavibacter* sp*.*** | **Sh2141*** | 90.37 | 88.78 | 88.76 | 89.63 | 89.07 | 90.60 | 90.59 | 90.77 |  | 37.30 | 36.90 | 37.10 | 37.50 | 37.60 | 39.50 | 38.50 | 39.00 | 36.20 | 40.20 |
| **10** | ***C. michiganensis*** | **LMG 7333^T^** | 90.23 | 89.29 | 89.16 | 89.71 | 89.71 | 89.71 | 89.62 | 89.73 | 89.29 |  | 57.10 | 45.90 | 48.60 | 48.00 | 49.50 | 44.90 | 40.20 | 37.50 | 37.90 |
| **11** | ***C. californiensis*** | **CFBP 8216^T^** | 90.07 | 89.15 | 89.07 | 89.52 | 89.41 | 89.63 | 89.49 | 89.53 | 89.14 | 94.07 |  | 45.20 | 47.00 | 46.30 | 47.30 | 44.00 | 39.30 | 37.10 | 37.80 |
| **12** | ***C. sepedonicus*** | **ATCC 33113^T^** | 89.95 | 88.80 | 88.61 | 89.15 | 89.16 | 89.41 | 89.24 | 89.35 | 89.04 | 91.67 | 91.52 |  | 45.10 | 45.20 | 46.60 | 43.90 | 39.10 | 36.40 | 37.30 |
| **13** | ***C. insidiosus*** | **LMG 3663^T^** | 90.44 | 89.35 | 89.15 | 89.63 | 89.74 | 89.74 | 89.66 | 89.80 | 89.34 | 92.29 | 92.15 | 91.52 |  | 59.90 | 48.3 | 45.50 | 40.50 | 37.80 | 38.40 |
| **14** | ***C. nebraskensis*** | **NCPPB 2581^T^** | 90.47 | 89.26 | 89.06 | 89.78 | 89.72 | 89.79 | 89.64 | 89.78 | 89.42 | 92.22 | 91.93 | 91.56 | 94.71 |  | 51.10 | 45.30 | 40.70 | 37.60 | 38.40 |
| **15** | ***C. phaseoli*** | **CFBP 8627^T^** | 90.46 | 89.43 | 89.39 | 90.23 | 89.54 | 89.98 | 89.93 | 84.16 | 89.63 | 92.74 | 91.89 | 91.45 | 92.45 | 92.55 |  | 40.90 | 43.30 | 40.00 | 40.7 |
| **16** | ***C. lycopersici*** | **CFBP 8615^T^** | 90.59 | 89.18 | 89.29 | 89.70 | 89.70 | 89.98 | 89.89 | 90.04 | 89.35 | 91.33 | 91.20 | 91.14 | 91.79 | 91.78 | 91.72 |  | 40.90 | 38.20 | 39.10 |
| **17** | ***C. capsici*** | **LMG 29047^T^** | 92.43 | 89.71 | 89.65 | 90.16 | 90.10 | 90.73 | 90.63 | 90.86 | 89.97 | 90.05 | 89.98 | 89.82 | 90.30 | 90.65 | 90.55 | 89.78 |  | 38.40 | 40.60 |
| **18** | ***C. tessellarius*** | **ATCC 33566^T^** | 90.01 | 92.79 | 92.71 | 98.02 | 98.08 | 90.02 | 89.88 | 90.06 | 89.79 | 89.38 | 89.35 | 88.91 | 89.69 | 89.84 | 89.54 | 88.93 | 90.06 |  | 38.00 |
| **19** | ***C. zhangzhiyongii*** | **LMG 31970^T^** | 90.90 | 89.49 | 89.41 | 90.12 | 89.99 | 97.90 | 97.97 | 97.34 | 90.47 | 89.28 | 89.29 | 89.20 | 89.74 | 89.80 | 89.74 | 89.24 | 90.63 | 90.09 |  |
