# Supplementary material for: The center of wheat domestication drives diversity of Clavibacter pathogens
Source: Appl Environ Microbiol. 2025 Oct 8;91(11):e01245-25. doi: 10.1128/aem.01245-25 (PMC12628682; doi:10.1128/aem.01245-25)
Supplement: Table S2 — Primer pairs used in this study. [file aem.01245-25-s0003.docx]

Table S2: Primer pairs used in this study.

| Primer name | 5′-3′ Sequence | Size of amplicon (bp) | Annealing temperature  (°C) | Target | Refrence |
| --- | --- | --- | --- | --- | --- |
| CMR16F1  CMR16R1 | GTGATGTCAGAGCTTCCTCTGGCGGATA  GTACGGCTACCTTGTTACGACTTAGT | 1425 | 62 | *Clavibacter* spp. | Lee *et* *al*., 1997 |
| CM3  CM4 | CCTCGTGAGTGCCGGGAACGTATCC  CCACGGTGGTTGATGCTCGCGAGAT | 639 | 60 | *C. michiganensis sensu lato* | Sousa-Santos et al., 1997 |
| atpD2F  atpD2R | GACATCGAGTTCCCGCAC  CGATGATCTCCTGGAGCTCCTTGT | 1104 | 55 | *atpD* | Jacques *et al*., 2012 |
| 2F  6R | ACCGTCGAGTTCGACTACGA  AGSACGATCTTGTGGTA | 977 | 57 | *gyrB* | Richert *et al*., 2005 |
| ppkF  ppkR | GAGAACCTCATCCAGGCCCT  CGAGCTTGCAGTGGGTCTTGAG | 604 | 60 | *ppk* | Jacques *et al*., 2012 |
| recAF  recAR | GACCGCGCTCGCACAGATCGACCG  GCCATCTTGTTCTTGGACGACCTTG | 724 | 63 | *recA* | Jacques *et al*., 2012 |
